# Supplementary material for: Synergistic Activity of DNA Damage Response Inhibitors in Combination with Radium-223 in Prostate Cancer
Source: Cancers (Basel). 2024 Apr 15;16(8):1510. doi: 10.3390/cancers16081510 (PMC11048209; doi:10.3390/cancers16081510)
Supplement: Supplementary file 1 [file cancers-16-01510-s001.zip › cancers-2916083-supplementary.pdf]

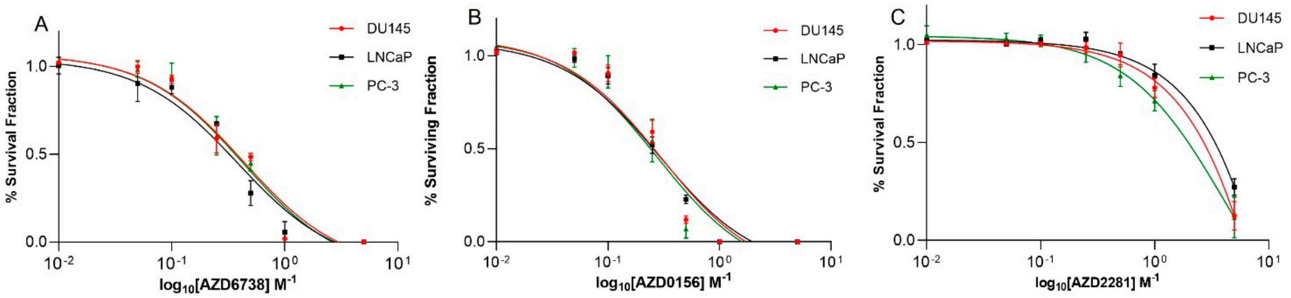

Supplementary Figure S1:

Cellular toxicity of the ATM inhibitor AZD0156 (A), the ATR inhibitor AZD6738 (B), and the PARP inhibitor AZD2281 (C) in human prostate cancer cell lines was determined using clonogenic survival assays. Cells were treated with a dose range of 0.01 µM - 5 µM. After 7-10 days of culture, colonies were counted and survival fractions calculated. Each value represents the mean from three independent experiments and the respective standard error.

DDR Inhibitor	Cell Line	IC_{50} (µM)
AZD0156	PC-3	0.28
	DU145	0.30
	LNCaP	0.29
AZD6738	PC-3	0.43
	DU145	0.43
	LNCaP	0.38
AZD2281	PC-3	4.24
	DU145	27.44
	LNCaP	50.64

Supplementary Table S1:

IC50 values of clonogenic survival assays for human prostate cancer cell lines indicating the DDR inhibitor concentration at which survival was reduced by 50%

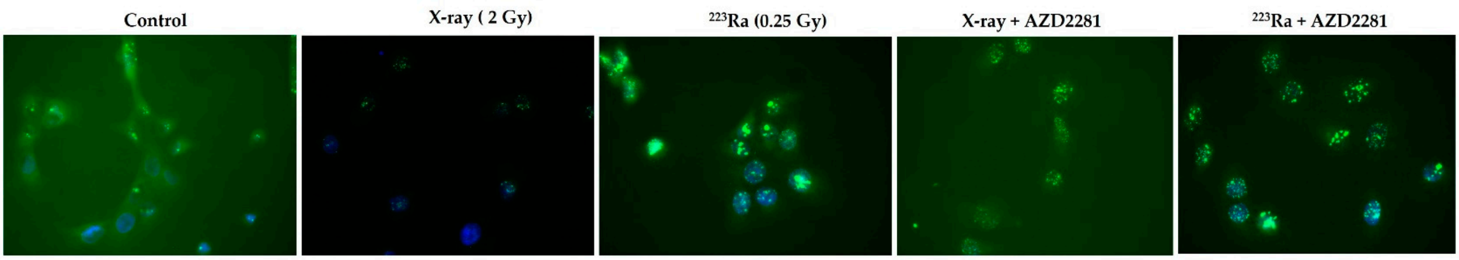

Supplementary Figure S2:

Representative images of mean 53BP1 foci per cell for DU145 human prostate cancer cell line following X-rays (2 Gy) ^{223}Ra (0.25 Gy) alone or in combination with AZD2281 (500 nM) at a 1 h timepoint.

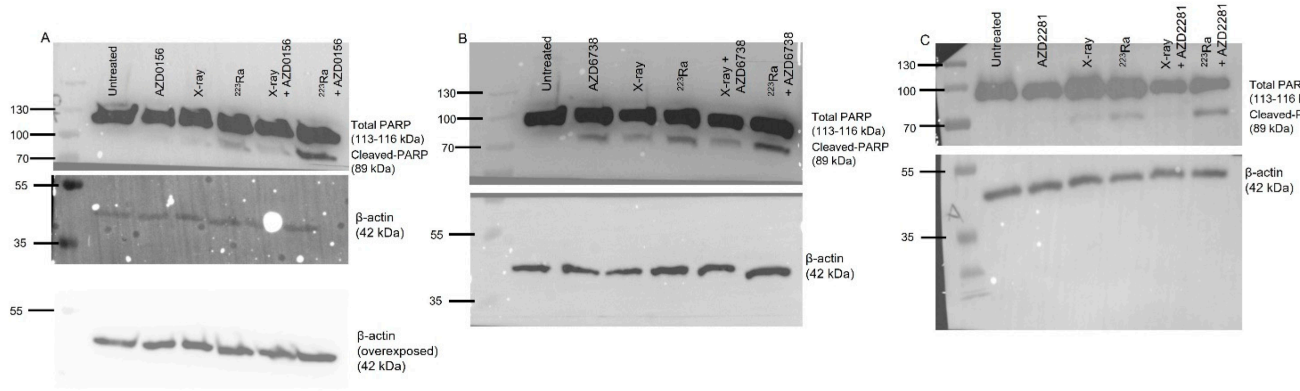

Supplementary Figure S3:

Whole western blots including molecular weight markers for protein expression for full and cleaved PARP-1 in the PC-3 human prostate cancer cell line at 48 h after treatment with X-rays (2 Gy), ^{223}Ra (0.25 Gy), AZD0156 (100 nM) (**A**), AZD6738 (100 nM) (**B**), or AZD2281 (500 nM) (**C**) alone or in combination. β-actin was used as a loading control. Figure A bottom blot is over-exposed, ladder is very faint.
